# Treatment of intramuscular lipoma of tongue with enveloped mucosal flap design: a case report and review of the literature

**DOI:** 10.1186/s40902-020-00281-4

**Published:** 2020-11-23

**Authors:** Sung-Hwi Hur, Jae-Seok Lim, Sun-Gyu Choi, Ji-Yeon Kang, Ji-Hye Jung, Eun-Young Lee

**Affiliations:** 1 Department of Oral & Maxillofacial Surgery, Hankook General Hospital, Cheongju, Korea; 2 Department of Oral & Maxillofacial Surgery, Chungbuk National University Hospital, Chungdae-ro 1, Seowon-Gu, Cheongju, Chungbuk 28644 Korea; 3 Department of Oral & Maxillofacial Surgery, College of Medicine, Chungnam National University, Daejeon, Korea; 4 Department of Oral & Maxillofacial Surgery, College of Medicine and Medical Research Institute Chungbuk, National University, Chungdae-ro 1, Seowon-Gu, Cheongju, Chungbuk 28644 Korea

**Keywords:** Lipoma, Tongue, Intramuscular lipoma, Excision, Enveloped mucosal flap

## Abstract

**Background:**

Lipomas are benign soft tissue neoplasms of mature adipose tissue commonly occurring in the trunk or extremities. But, intraoral lipomas are rare entities which may be only noticed during routine dental examinations. Especially intramuscular lipomas on the tongue have been reported very rarely. In this study, we report a case of intramuscular lipoma on tongue, with a review of the literature from 1978 to 2019, providing data on age, gender, location, presenting symptoms, size, surgical methods, and recurrence.

**Case presentation:**

A case of intramuscular lipoma occurring in tongue region in a 65-year-old male is reported. Surgical excision is the mainstay of treatment for the lesion. In order to decrease the deformity and discomfort after the excision, we tried to modify surgical technique using enveloped mucosal flap. This technique provided more comfortable healing procedure on the operative site without recurrence.

**Conclusion:**

This is a rare case of large intramuscular lipoma on tongue. Surgical excision with enveloped mucosal flap design was performed to diminish postoperative raw surface and discomfort and a 24-month follow-up showed excellent healing without any recurrence. A case of intramuscular lipoma on tongue and relevant literature reviews are presented in this study.

## Introduction

Lipoma is the most common benign tumor seen as a common entity in the trunk or extremities, accounting for 50% of all soft tissue neoplasm [1, 2]. It occurs in about 15–20% on head and neck region, but oral lipomas are uncommon in general, representing approximately 0.2–5% of all benign neoplasms of the oral cavity [1, 3–10]. In the oral cavity, lipoma on tongue is very rare. It occurs only 0.3% of all tongue tumors [6, 11] When lipomas occur intraorally, the buccal mucosa is the most frequent site of occurrence, followed by tongue [8, 10].

Soft-tissue lipomas can be categorized into two subtypes. Usually, lipomas arise in subcutaneous tissues (superficial lipomas) [2]. But, there are unusual deep-seated subtypes, which are localized deep in muscle; known as intramuscular lipoma or infiltrating lipoma [12, 13]. They rarely occur, accounting for less than 1% of all lipomas [14]. Taken together, intramuscular lipomas on tongue are exceedingly rare, with few reported cases worldwide in English literatures (Table 1). They account for only 3%-7% of oral lipomas [7, 9].

**Table 1:** Epidemiologic and Clinical features of intramuscular lipoma on tongue reported in English in 1978-2019 (including present case)

Author	Year	Age/sex	Site(of tongue)	Symptom	Max. dia	Tx.	F/U	Rec
Bennhoff DF [15]	1978	68/M	Rt. lateral side	Swelling, painless	NA	Excision	NA	NA
Garavaglia J [16]	1987	38/M	Rt. lateral side	Swelling, painless	1.5	Excision	25	No
Shirasuna K [17]	1988	56/F	Ventral surface	Swelling, painless	1.8	Excision	12	No
Takeda Y [18]	1989	37/M	NA	Swelling, painless	4.0	Excision	NA	NA
Kacker A [19]	1996	78/M	Rt. lateral side	Swelling, painless	6.0	Excision	NA	NA
Epivatian A [20] (2case)	2000	64/F 56/F	Dorsal surface Dorsal surface	Swelling, painless Swelling, painless	2.5 3.0	Excision Excision	60 6	No No
Thomas S [21]	2002	42/M	both lateral side	Swelling, painless, impaired speech	4.0(Rt.) 3.0(Lt.)	Excision	18	No
Keskin G [22]	2002	54/M	both lateral side	Swelling, painless	1.0(both)	Excision	13	No
Colella G [23]	2004	54/M	Lt. lateral side	Swelling, painless	2.5	Excision	6	No
Akbulut M [24]	2005	50/F	Rt. lateral side	Swelling, painless	0.6	Excision	60	No
Bandeca MC [25]	2007	62/F	Ventral surface	Swelling, painless	5.0	Excision	8	No
Colella G [26]	2009	75/M	Anterior tip	Swelling, painless	10.0	Excision	15	No
Garg M [27]	2011	55/M	Lt. lateral side	Swelling, painful, impaired deglutition	1.0	Excision	NA	NA
Naruse T [28]	2012	58/F	Lt. lateral side	Swelling, painless	3.5	Excision	15	No
Amirzadeh A [29]	2013	68/M	Anterior tip	Swelling, painless, Impaired speech	NA	Excision	NA	NA
Saxena S [30]	2014	50/F	Rt. lateral side	Swelling, painless	2.0	Excision	12	No
Sudha SM [31]	2014	75/M	Lt. lateral side	Swelling, painless	1.5	Excision	NA	NA
Prabhala S [32]	2015	75/M	Lt. lateral side	Swelling, painless	1.8	Excision	4	No
Namboodiripad A [33]	2016	60/F	Ventral surface	Swelling, painless	1.0	Excision	6	No
Kohinata K [34]	2018	62/M	Ventral surface	Swelling, painless	2.0	Excision	NA	No
Fitzgeralda K [35]	2018	57/M	Rt. lateral side	Swelling, painless	4.4	Excision	10	No
Monda K [36]	2019	68/F	Lt. lateral side	Swelling, painless	4.0	Excision	12	No
Present case	–	65/M	Rt. lateral side	Swelling, painless, impaired speech	4.0	Excision	12	No

*Age years *Max. dia maximum diameter, cm,*F/U follow-up, month, *Rec recurrence, *NA not available

Commonly, surgical excision has been considered for the treatment method of lipoma. However, when the size of the lesion is large, it is likely to cause aesthetic side effects due to the defects after surgical excision. Surgical excision with envelope flap design was performed to diminish postoperative raw surface and discomfort. Here, we report a case of intramuscular lipoma of the tongue which was treated by surgical excision with the envelope technique and the review of literature. A Google Scholar search for the terms “intramuscular lipoma,” “infiltrating lipoma,” “tongue,” and “lingual” within a period from 1978 to 2019 was performed, total 22 literatures published in English [15–36].

## Case presentation

**Fig. 1:**
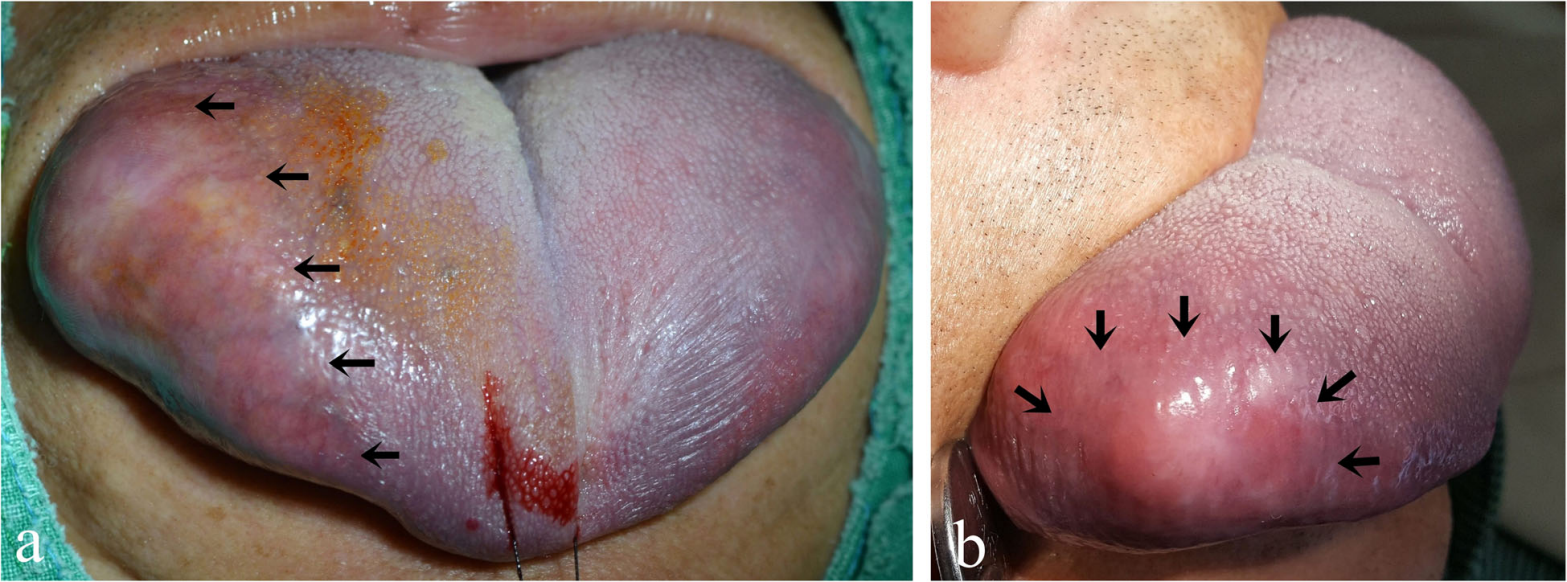
**a**, **b**. A painless mass on the right lateral border of tongue

A 65-year-old male nonsmoker presented with a painless mass on the right lateral side of his tongue (Fig. 1). This lesion developed from 4 to 5 months ago and became increasingly larger in size. The patient had not previously noticed the mass and did not complain of any functional impairment. However, speech was slightly impaired. Physical examination revealed swelling on tongue, but other aspects of the examination were unremarkable. The mucosal covering of the lesion appeared clinically normal. It was doughy in consistency, not tender, and no pulsations were felt on palpation. The patient’s personal and family history was also unremarkable. The preliminary differential diagnosis was determined to be angioedema, neurofibroma, lipoma, fibrolipoma, glandular cell tumor, schwannoma, or dermoid cyst of tongue.

**Fig. 2:**
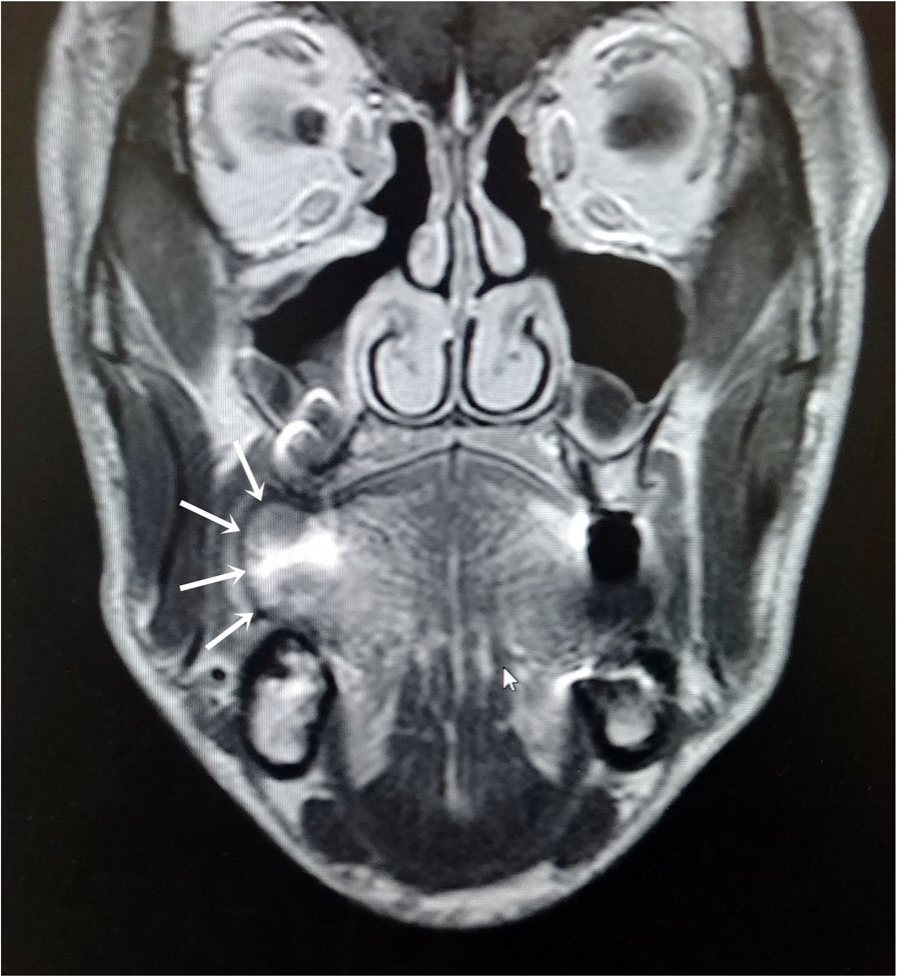
MRI showing a submucosal swelling within the muscles of the tongue on Rt. lateral side (white arrows)

Magnetic resonance imaging (MRI) showed a 2 cm × 4 cm well-remarked lesion within the muscles of the tongue. The findings were reported as consistent with lipoma (Fig. 2).

**Fig. 3:**
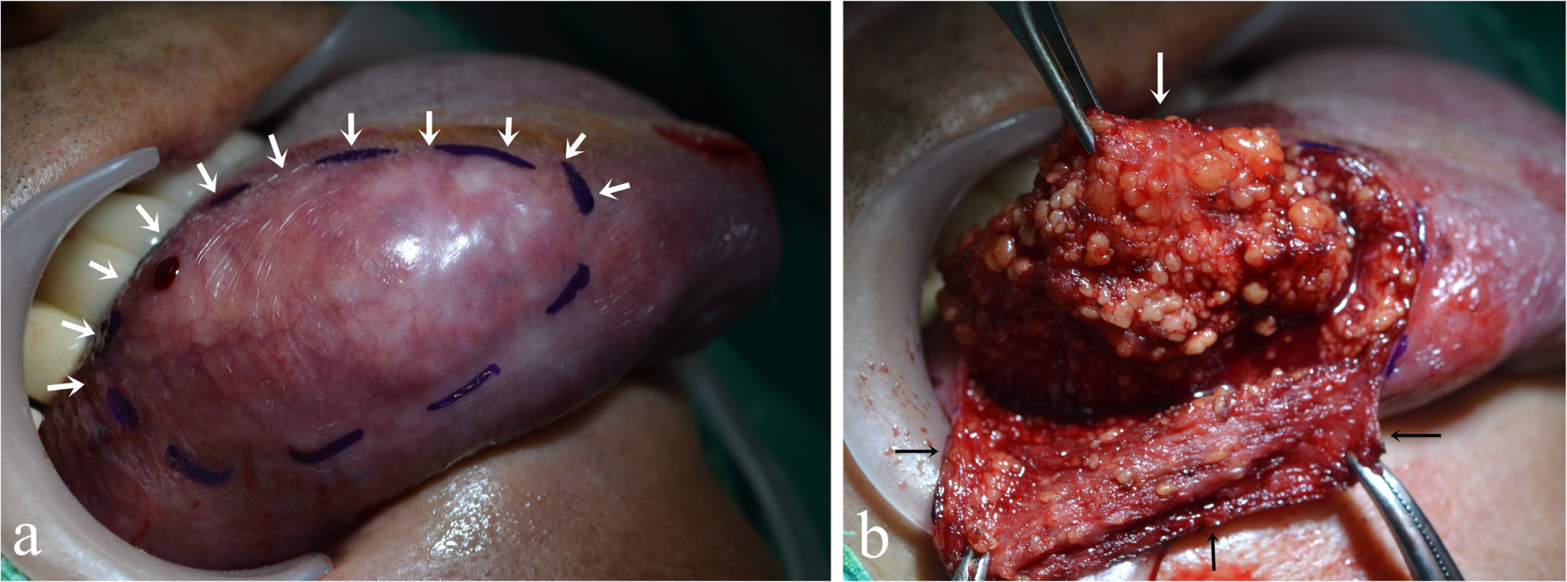
**a** The flap was designed as an envelope form (incision line-white arrows). **b** Surgical excision toward muscle side was done (white arrow). Then, the preserving mucosal flap was sutured to its original position (black arrows)

**Fig. 4:**
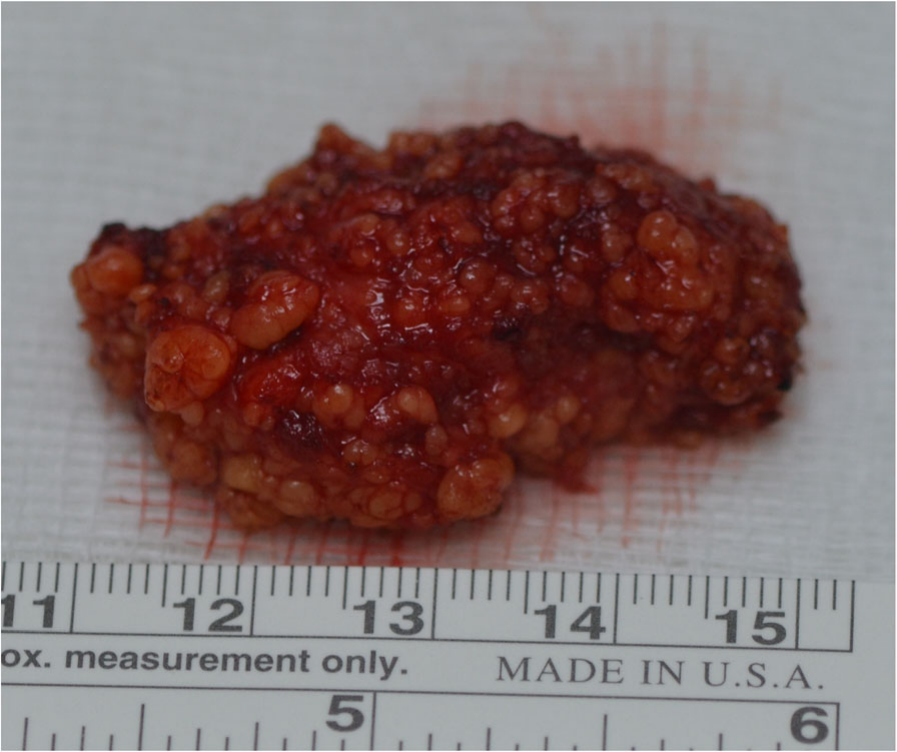
The size of the tumor was 2 × 4 × 1.5 cm, surrounded by 0.1–0.3 cm yellow fat granules

First, incisional biopsy was performed and it was diagnosed as lipoma. Then complete resection of the lesion and biopsy were performed under the general anesthesia. For preservation of superficial mucosa and avoid of raw area, the enveloped flap was designed (Fig. 3). The surrounding tissues surrounded by membranes, and penetrated the surrounding muscle tissue, and the boundary was not clear. Surgical excision with a surrounding rim of normal tissue was done. The surgical profile of remained tongue was confirmed within normal limits by the intraoperative frozen biopsy. The mass revealed a non-encapsulated, spherical, yellowish, solid, and greasy mass 2 × 4 × 1.5 cm in size (Fig. 4).

**Fig. 5:**
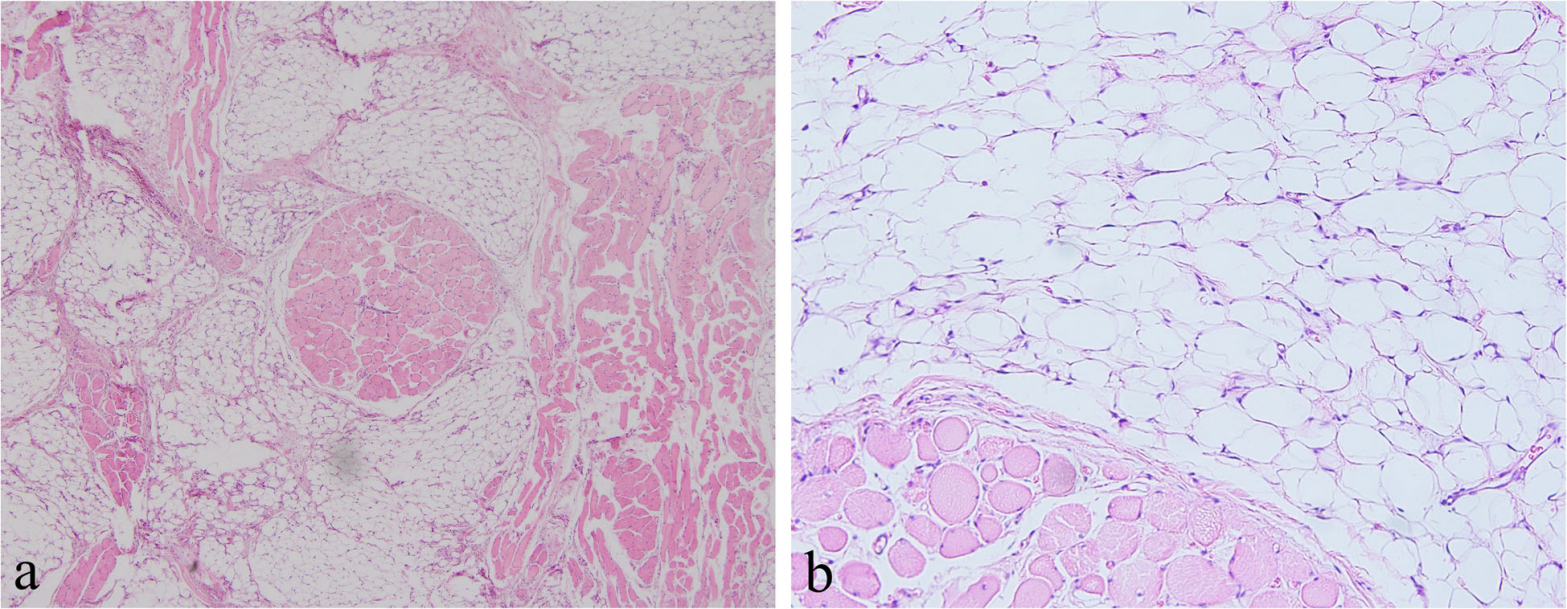
Histopathological features of the mass. **a** Adipocytes are seen diffusely infiltrating adipocytes the skeletal muscle (H and E stain, original magnification × 50). **b** Microphotograph showing mature fat cells with nuclei peripherally located (H and E stain, original magnification × 200)

Microscopic examination revealed multiple lobulated sections of mature, univaculated adipocytes of relatively uniform in size and shape with occasional entrapped skeletal muscle fibers. There was no evidence of hyperchromasia, pleomorphism, or multi-nucleation of adipocytes and no evidence of lipoblasts. The entrapped muscle fibers usually show few changes other than various degrees of muscular atrophy (Fig. 5). The histopathology was consistent with the final diagnosis of intramuscular lipoma.

**Fig. 6:**
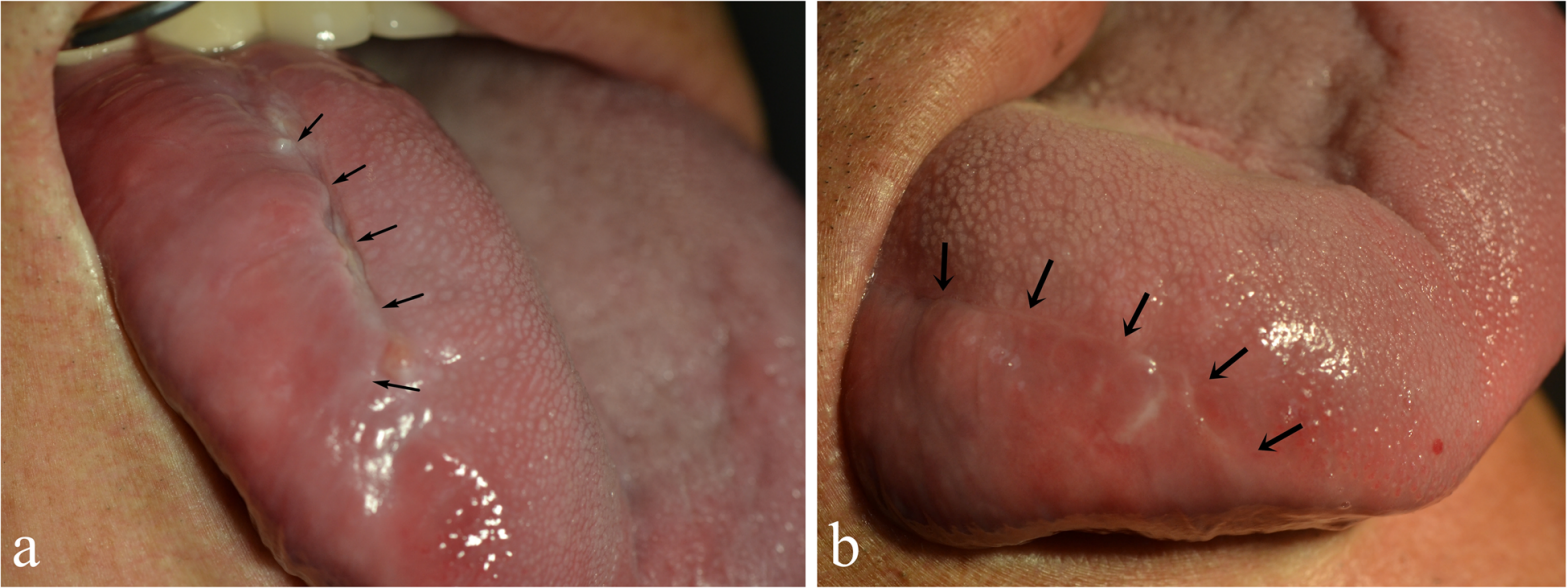
**a** Tumor was surgically excised with restoration of normal tongue function and aesthetic appearance. Two weeks after the operation, healed well without any complications. **b** After 3 months, the aesthetic appearance of the tongue was well restored and no recurrence was seen

The postoperative period was uneventful. There were no other symptoms such as pain, sensory disturbance, nor movement disorder other than discomfort. The patient’s tongue function and appearance were normal (Fig. 6). There was no evidence of recurrence during a 24-month follow-up period.

Written informed consent was obtained from the patient for publication of this case report and accompanying images (IRB No. 2020-07-022).

## Discussion

Lipomas are common soft tissue benign tumors, but are rare in the oral cavity, and especially on tongue. The incidence is very low [3–11]. Infrequently, lipomas can arise inside the muscle and are called ‘intramuscular(infiltrating) lipoma’. In 1853, muscular lipoma was first reported by Paget et al. described a lipoma infiltrating into the trapezius muscle. In 1946, the term of “infiltrating lipoma” was first defined in Regan’s article, referring to a lipoma infiltrating into muscle [37]. It is reported that this rare type of lipoma accounts for less than 1% of all lipomas [14]. The tongue lesion reported in our case was diagnosed as intramuscular lipoma. This case is unique because it is a very rare subtype of lipoma occurring in a very rare site.

In order to analyze the epidemiologic and clinical features of intramuscular lipoma developed on tongue, we have searched the literature published in English so far by Google Scholar and reviewed all case reports including our case (Table 1).

Since the first intramuscular lipoma on tongue was reported by Bennhoff et al. in 1978, the total of 24 cases have been reported in 23 literatures in English, including present case. Age distribution was shown from 37 to 75 years, and most of the patients over 50 years old (only 3 cases under 50 years). With regard to gender distribution, prevalence of the males was almost twice as high as that of females with 15 cases for male and 9 cases for female. As for the site of occurrence, the lateral side of the tongue was the majority with 16 cases including our case, followed by ventral surface with 4 cases, dorsal surface with 2 cases, and anterior tip with 2 case. The lesion size varied from 0.6 to 10 cm in maximum diameter with an average of 3.18 cm. Because the lesions are typically round or fusiform in shape, the size was analyzed based on the maximum diameter. In most cases, the patient had asymptomatic, painless swelling lesions, but in one case, painful swelling and difficulty in deglutition were present. In three cases, speech difficulties were found. Excision was performed as a treatment method in all cases. Except for six cases without information in the literature, there are no recurrences after surgery in all other cases during the average follow-up period of 17.29 months. Most of the features were not significantly different from simple lipoma of the oral cavity, but in gender distribution, intramuscular lipoma on the tongue was more prevalent in male, whereas simple lipoma was higher in female [8].

Clinically, intramuscular lipomas present as slow-growing, painless, rubbery, and swelling masses. Pain is an uncommon symptom. As the tumor grows in size, functional limitations can arise. Especially on tongue, it can cause discomfort on speech, deglutition, or mastication [12, 21, 25, 27, 29].

The exact etiology of intramuscular lipoma is still unclear. Trauma and chronic irritation have been suggested as probable causes, and also genetic factors and hormonal imbalance are suggested to be related [13, 38, 39].

Imaging such as the computed tomography (CT) scan or MRI plays an important role in making a diagnosis of intramuscular lipoma. In addition, preoperative imaging analysis is essential for defining the size, border, and location of the mass, and relationship with surrounding anatomical structures. Based on them, a surgical plan should be established. Imaging features on CT scan of intramuscular lipoma indicate a lesion situated within the muscle and thick and thin soft tissue density streaks inside a fat density lesion. MRI is also useful method for identifying lipomatous lesions. On MRI, the adipose tissue in the intramuscular lipomas appears as strikingly high intensity signal area on both T1- and T2-weighted images [12, 40, 41]. In our case, a preoperative diagnosis of lipoma was suggested on MRI features.

Intramuscular lipomas have typical histological features. They are histologically well-demarcated but unencapsulated, and have mature uni-vacuolated adipocytes irregularly infiltrating adjacent muscle fibers. No lipoblasts are identified. Also there is no increased mitosis, nuclear atypia, pleomorphysim, cellular hyperchromatism, and lipoblastic proliferation. They show the vasculature composed of thin-walled capillaries, which is occasionally not observed due to compression by surrounding adipocytes [12, 35, 41].

Meanwhile, what should be noted about intramuscular lipomas is that their clinical, histological and imaging characteristics are similar to well-differentiated liposarcomas, malignant tumors. This makes the differential diagnosis difficult [12, 42]. Therefore, careful clinical, histological, and imaging examinations are all necessary for accurate differential diagnosis of intramuscular lipomas, not just one examination alone. For the differential diagnosis in our case, clinical examination, MRI taking, and preoperative biopsy were all performed.

There are differences in imaging features between liposarcomas and intramuscular lipomas. On CT scans, liposarcomas, unlike intramuscular lipomas described above, are characterized by lesions spreading in the intra- and intermuscular layers and fat density lesions with a fair hazy amorphous areas and soft tissue density septa. And they are more oval shaped compared to intramuscular lipomas [40]. On MRI, liposarcomas tend to be larger than intramuscular lipomas; however, size alone is not a good predictive factor for malignancy. In contrast to intramuscular lipomas, liposarcomas are usually multilobular and have more and thicker septae with nodules [38]. The most distinctive histological point of liposarcomas is the presence of lipoblasts. And different from intramuscular lipoma, they exhibit features of increasing mitosis, mixoid differentiation, cellular pleomorphism, lipoblastic proliferation, and abundant vascularity [12, 24].

Due to their infiltrating tendency, the recurrence rate of intramuscular lipomas is known to be higher than that of other subtypes of lipoma. The recurrence rate after treatment ranges from 3 to 62.5% depending on the researchers [10, 13, 27, 40, 43]. However, in the literature we reviewed, there were no recurrences in all patients except for a few without information on recurrence (Table 1). All clinicians, including us, surgically treated patients with intramuscular lipomas on tongue and no local recurrence was noticed. Like other lipomatous tumors, the most main treatment of these lesions is surgical excision. Marginal excision of the well-circumscribed area and wide excision with free margin in the infiltrative areas are performed to prevent recurrence. But its condition infiltrating into the muscle fiber makes complete excision difficult [42, 44]. Conservative treatment is also practiced, but its role is very limited. Although there have been reports of animal studies that steroid injection into the lesion was effective, the efficacy in humans has not been proven [45].

When the lesion is large, surgical excision on the tongue lesion can result in the large defect, delayed healing, and patient discomfort. Primary suture was difficult and morphological deformation was predicted due to the large lesion. In this case, we opted for the enveloped flap design because the lesion was 4 cm large. In order to diminish the patient discomfort and reduce healing period, we used surgical excision with enveloped mucosal flap (Fig. 3b). Enveloped flap technique with checked margin free is advantageous in terms of surgical field of view, healing time and postoperative esthetics when extensively excising a large lesion. Extensive muscle resection and the intraoperative biopsy were performed to prevent recurrence. All adipose tissues of mucous membrane were removed and then sutured. The raw surface of tongue was minimized to reduce the healing period (Fig. 6).

## Conclusion

Although very rare, intramuscular lipomas can arise on tongue. Adequate preoperative examinations for accurate differential diagnosis, especially well-differentiated liposarcoma, and appropriate surgery are required to prevent recurrence. In our case, we performed MRI, biopsy before operation. Surgical excision with envelope flap design was performed to diminished postoperative raw surface and discomfort, and a 24-month follow-up showed excellent healing without any deformation and malfunction. Recurrence was not observed for a follow-up period, but long-term follow-up should be required.

## Data Availability

Not applicable

## Abbreviations

CT: Computed tomography
MRI: Magnetic resonance imaging

## References

[1] Fletcher CD, Unni KK, Mertens F (Eds.) (2002) Pathology and genetics of tumours of soft tissue and bone (Vol. 4). Iarc.

[2] Murphey MD, Carroll JF, Flemming DJ, Pope TL, Gannon FH, Kransdorf MJ (2004). From the archives of the AFIP: benign musculoskeletal lipomatous lesions. Radiographics.

[3] de Visscher JG (1982). Lipomas and fibrolipomas of the oral cavity. J Maxillofac Surg.

[4] Kumaraswamy SV, Madan N, Keerthi R, Shakti S (2009). Lipomas of oral cavity: case reports with review of literature. J Maxillofac Oral Surg.

[5] Kumar LK, Kurien NM, Raghavan VB, Menon PV, Khalam SA (2014) Intraoral lipoma: a case report. Case reports in medicine, 2014. 10.1155/2014/480130 PMC3926394 24592278

[6] Baonerkar HA, Vora M, Sorathia R, Shinde S (2015). The lipoma of tongue-A rare site for a tumor: Case report and review of the literature. Indian J Dent.

[7] Egido-Moreno S, Lozano-Porras AB, Mishra S, Allegue-Allegue M, Marí-Roig A, López-López J (2016). Intraoral lipomas: review of literature and report of two clinical cases. J Clin Exp Dent.

[8] Naruse T, Yanamoto S, Yamada SI, Rokutanda S, Kawakita A, Takahashi H (2015). Lipomas of the oral cavity: clinicopathological and immunohistochemical study of 24 cases and review of the literature. Indian J Otolaryngol Head Neck Surg.

[9] Manor E, Sion-Vardy N, Joshua BZ, Bodner L (2011). Oral lipoma: analysis of 58 new cases and review of the literature. Ann Diagn Pathol.

[10] Fregnani ER, Pires FR, Falzoni R, Lopes MA, Vargas PA (2003). Lipomas of the oral cavity: clinical findings, histological classification and proliferative activity of 46 cases. Int J Oral Maxillofac Surg.

[11] Chung JCK, Ng RWM (2007). A huge tongue lipoma. Otolaryngol Head Neck Surg.

[12] McTighe S, Chernev I (2014) Intramuscular lipoma: a review of the literature. Orthop Rev 6(4) 10.4081/or.2014.5618 PMC4274454 25568733

[13] Ramos-Pascua LR, Guerra-Álvarez OA, Sánchez-Herráez S, Izquierdo-García FM, Maderuelo-Fernández JÁ (2013). Intramuscular lipomas: Large and deep benign lumps not to be underestimated. Review of a series of 51 cases. Rev Esp Cir Ortop Traumatol.

[14] Myhre-Jensen O (1981). A consecutive 7-year series of 1331 benign soft tissue tumours: clinicopathologic data. Comparison with sarcomas. Acta Orthop Scand.

[15] Bennhoff DF, Wood JW (1978). Infiltrating lipomata of the head and neck. The Laryngoscope.

[16] Garavaglia J, Gnepp DR (1987). Intramuscular (infiltrating) lipoma of the tongue. Oral Surg Oral Med Oral Pathol.

[17] Shirasuna K, Saka M, Watatani K, Kogo M, Matsuya T (1989). Infiltrating lipoma of the tongue. Int J Oral Maxillofac Surg.

[18] Takeda Y (1989). Intramuscular lipoma of the tongue: report of a rare case. Ann Dent.

[19] Kacker A, Taskin M (1996). Atypical intramuscular lipoma of the tongue. J Laryngol Otol.

[20] Epivatianos A, Markopoulos AK, Papanayotou P (2000). Benign tumors of adipose tissue of the oral cavity: a clinicopathologic study of 13 cases. J Oral Maxillofac Surg.

[21] Thomas S, Varghese BT, Sebastian P, Koshy CM, Mathews A, Abraham EK (2002). Intramuscular lipomatosis of tongue. Postgraduate medical journal.

[22] Keskin G, Ustundag E, Ercin C (2002). Multiple infiltrating lipomas of the tongue. J Laryngol Otol.

[23] Colella G, Lanza A, Rossiello L, Rossiello R (2004). Infiltrating lipoma of the tongue. Oral Oncol Extra.

[24] Akbulut M, Aksoy A, Bir F (2005). Intramuscular lipoma of the tongue: a case report and review of the literature. Aegean Pathology Journal.

[25] Bandéca MC, De Pádua JM, Nadalin MR, Ozório JEV, Silva-Sousa YTC, Perez DEDC (2007) Oral soft tissue lipomas: a case series. J Can Dent Assoc 73(5) 17555654

[26] Colella G, Biondi P, Caltabiano R, Vecchio GM, Amico P, Magro G (2009). Giant intramuscular lipoma of the tongue: a case report and literature review. Cases J.

[27] Garg M, Aggarwal R, Sethi D, Gupta D, Sen R (2011). Intramuscular lipoma of tongue. J Cutan Aesthet Surg.

[28] Naruse T, Yanamoto S, Kawano T, Yoshitomi I, Yamada SI, Kawasaki G (2012). Intramuscular lipoma of the tongue: Report of a case complicated with diffuse lipomatosis. J Oral Maxillofacial Surg, Med Pathol.

[29] Amirzadeh A, Klaustermeyer W (2013). Intramuscular lipoma of the tongue masquerading as angioedema. Ear, Nose Throat J.

[30] Saxena S, Jahagirdar P, Chidananda D (2014). Infiltrating oral lipoma a rare variant. J Cutan Aesthet Surg.

[31] Sudha SM, Subramanyeshwar RT, Sufith CP (2014) Intramuscular lipoma of the tongue: A rare site for a common tumour. Case Reports in Clinical Pathology, Vol. 1, No. 1.

[32] Prabhala S, Jayashankar E, Reddy MS, Tanikella R (2015) Intramuscular lipoma of tongue: A common tumor at an uncommon site. Medical Journal of Dr. DY Patil University, 8(5), 656.

[33] Namboodiripad AP, Kalliath R, Chammanam SG, Nair A, Rachana PB, Divya R (2016) An Intramuscular Lingual Lipoma: A Case Report and Review. Oral & Maxillofacial Pathology Journal 7(2)

[34] Kohinata K, Uchida K, Ochiai T, Kroiwa H, Yamada S, Sugino N (2018). A Case of Intramuscular Lipoma Arising in the Inferior Surface of the Tongue. Int J Dent & Oral Heal.

[35] Fitzgerald K, Sanchirico PJ, Pfeiffer DC (2018). Large intramuscular lipoma of the tongue. Radiol Case Rep.

[36] Mondal K, Mandal R (2019). Cytological diagnosis of an intramuscular lipoma located on the tongue. Int J Health Allied Sci.

[37] Regan JM (1946). Infiltrating benign lipomas of the extremities. West. J. Surg. Obstet. Gynecol..

[38] Pichierri A, Marotta N, Raco A, Delfini R (2010). Intramuscular infiltrating lipoma of the longus colli muscle. a very rare cause of neck structures compression. Cent Eur Neurosurg.

[39] Copcu E (2003). Can intramuscular lipoma have a post-traumatic origin?. Brit J Dermatol.

[40] Nishida J, Morita T, Ogose A, Okada K, Kakizaki H, Tajino T (2007). Imaging characteristics of deep-seated lipomatous tumors: intramuscular lipoma, intermuscular lipoma, and lipoma-like liposarcoma. J Orthop Sci.

[41] Rougraff BT, Durbin M, Lawerence J, Buckwalter K (1997). Histologic correlation with magnetic resonance imaging for benign and malignant lipomatous masses. Sarcoma.

[42] Han HH, Choi JY, Seo BF, Moon SH, Oh DY, Ahn ST, Rhie JW (2014, 2014) Treatment for intramuscular lipoma frequently confused with sarcoma: a 6-year restrospective study and literature review. Biomed Res Int 10.1155/2014/867689 PMC4276111 25574469

[43] Dionne GP, Seemayer TA (1974). Infiltrating lipomas and angiolipomas revisited. Cancer.

[44] Su CH, Hung JK, Chang IL (2011). Surgical treatment of intramuscular, infiltrating lipoma. Int Surg.

[45] Lamagna B, Greco A, Guardascione A, Navas L, Ragozzino M, Paciello O (2012). Canine lipomas treated with steroid injections: clinical findings. PloS one.

